# Medical Opinion and Movement

**Published:** 1907-05-04

**Authors:** 


### Medical Opinion and Movement.

The Conference, which we announced in a previous issue, on the teaching of hygiene and temperance in the universities and schools of the British Empire, took place in the Examination Hall, Victoria Embankment, on St. George's Day, April 23rd. Lord Strathcona opened the proceedings in the morning and Sir John Gorst presided in the afternoon. Among those who also took part in the proceedings were Mr. Deakin, the Australian Premier, Sir Victor Horsley, Sir Lauder Brunton, Sir Phillip Jones, and Sir John Cockburn. Several interesting addresses were delivered on the subject. Sir Victor Horsley referred especially to the question of alcoholism in relation to the secondary school. Investigations had enabled him to estimate that in at least 72 per cent. of these schools total abstinence was practised by the pupils, but there were still schools, mainly old-established public schools, in which the use of alcohol was not only connived at, but authorised. The outcome of the conference was expressed in two resolutions, which were carried unanimously; one urging the necessity of making hygiene and temperance an essential part in all educational systems, and the other expressing the opinion that it was essential that a medical department should be instituted in connection with the Board of Education.

With reference to the recent resignations at the Sheffield Union Hospital, to which we referred in our last issue, we are now informed that the details of the circumstances under which these resignations were accepted by the authorities have been incorrectly stated. Our information was derived, as stated at the time, from an article in the *Sheffield Independent*, and our remarks were prompted by the feeling that if the statements contained in that article were true, grave injustice had been done to the medical staff of the Union Hospital. Under the circumstances, we are glad to hear that the guardians "have sanctioned nothing at variance with the rule that servants of the hospital are under the control of the medical staff." At the same time, eight resignations, following close upon one another, prove that the relations between the resident medical staff and the local guardians are not such as they should be. The essentials of the efficient administration of a great hospital are apparently not understood by the guardians. No patient should be admitted, allocated to a ward, or removed from one ward to another without the order of the medical officer.

The proposal to impose an income-tax in France, if it becomes law, is likely to place the medical practitioner in a serious dilemma. The scheme as drawn up by M. Caillaux provides that the annual returns of profits from those of the liberal professions shall be accompanied by all vouchers necessary to establish their correctness. It is assumed that under these circumstances doctors may be called upon to produce their books and records of visits and consultations. In this way the comptroller will become cognisant of the names of patients and of the details of their maladies. By the penal code medical men are liable to imprisonment and to a fine to the extent of £25 for any disclosure of secrets entrusted to them in their professional capacity, except in cases where the law compels such disclosure. It may be that under the proposed circumstances it will be held that there is compulsion by the law. In that case such wholesale disclosures to a third person, even though he be sworn to secrecy, are likely to give rise to serious inconveniences. Moreover, there is to be a Control Committee appointed by the Prefect, which will also have the right to free access to all facts and figures. The matter has been freely discussed by the Congress of Medical Practitioners held recently in Paris, and has formed the subject of a resolution condemning the Bill.

In November last an international agreement was signed at Brussels by representatives from most of the countries of Europe, including Great Britain, for the unification of the strengths, formulæ, and processes of preparation of potent drugs. The text of the agreement has been recently published, and a report has been submitted to the Pharmacopœia Committee of the General Medical Council by the Committee of reference in pharmacy, showing the changes which will be necessary in the next issue of the British Pharmacopœia in order to give effect to this international agreement. Most of the changes are unimportant, and can be made without inconvenience. The tincture of belladonna is to be made from the dried leaves, not including the flowering tops, and the galenical preparations of hyoscyamus are also to be made only from the leaf. The changes of serious consequence are in the strength of certain preparations, and these are in some cases of such proportion as to require careful attention in order to avoid any serious mishaps. The tincture of strophanthus, for instance, will be increased nearly fourfold. The ethereal tincture of lobelia will be twice the present strength. On the other hand, the tincture of digitalis will be reduced by one-fourth, and the tincture of nux vomica by one-half. The committee point out that the standardisation of nux vomica is of a retrograde nature, as it is to be estimated according to the total alkaloid, instead of, as at present, according to the amount of active strychnine. The ratio of active strychnine to brucine is very variable, and a standardisation according to total alkaloid would not ensure the necessary presence of any strychnine. It is probable that in such cases the British Government will exercise the right provided in the agreement to make such modifications in detail as the progress of medical and pharmaceutical science may from time to time render necessary.

